# Parathyroidectomy reduces the costs of medication in patients with secondary hyperparathyroidism

**DOI:** 10.1016/j.clinsp.2024.100484

**Published:** 2024-09-14

**Authors:** Gabriel Mattucci Domingues Pereira, Matheus Liao, Sergio Samir Arap, Felipe Ferraz Magnabosco, Marilia D'Elboux Guimarães Brescia, Rosa Maria Affonso Moyses, Melani Ribeiro Custódio, Vanda Jorgetti, Luiz Paulo Kowalski, Fábio Luiz de Menezes Montenegro

**Affiliations:** Hospital das Clínicas da Faculdade de Medicina da Universidade de São Paulo, São Paulo, SP, Brazil

**Keywords:** Secondary hyperparathyroidism, Chronic kidney disease, Costs, Parathyroidectomy

## Abstract

**Introduction:**

Subtotal Parathyroidectomy (S-PTx) and total Parathyroidectomy with immediate Autograft (PTx-AG) are well-established techniques for the treatment of refractory Secondary Hyperparathyroidism (SHPT), with comparable improvements in patients' quality of life and survival. However, the long-term costs after these operations may impact the choice of surgical technique. The objective of the study is to analyze the impact of surgical treatment on medication costs and whether there is any difference between medication use after each procedure, considering impacts on the health system.

**Material and methods:**

Prospective and randomized study in patients with severe SHPT undergoing S-PTx and PTx-AG. Analysis of prescribed medication costs in the month before the postoperative period at 1-, 3-, 6-, 12-, and 18 months. Costs were estimated according to government payment system values. The medications of 65 patients after PTx-AG were compared with those of 24 patients after S-PTx. A comparison of the total costs of the period between 38 men and 51 women was also made.

**Results:**

There were 89 evaluable cases. Surgery reduced medication costs after 12 months. The median of total drug costs in the analyzed period was R$ 8,375.00 per patient. There was no difference in costs per patient in the S-PTx group compared to the PTx-AG group. The median total costs were R$ 11,063.0 for men and R$ 7,651.0 for women (p = 0.0078).

**Conclusions:**

The type of parathyroidectomy did not impact costs after surgery. In the first year after surgery, the use of calcium and calcitriol was more significant than the use of other medications. In the following months, the use of sevelamer is responsible for the highest costs. Men have higher costs in outpatient follow-up after surgery.

## Highlights

• Subtotal or Total Parathyroidectomy with autograft equally reduces the costs of medication in patients with secondary hyperparathyroidism in the long run.
• Endocrine surgeons could individualize the operation technique in the treatment of hyperparathyroidism in dialysis patients.
• The first month after parathyroidectomy shows an increased medication cost compared with the preoperative period in secondary hyperparathyroidism.

## Introduction

According to recent data, between 5 % and 10 % of the world's population has Chronic Kidney Disease (CKD). In Brazil, its incidence has increased due mainly to the increase in the longevity of the population and to the greater number of diagnosed patients.^1^ Among the metabolic alterations of CKD are hyperphosphatemia, decreased production of calcitriol by the kidney, and resultant hypocalcemia. From a more advanced stage, these parameters are responsible for the development of important complications, such as Secondary Hyperparathyroidism (SHPT),^2^ which affects approximately 44 % of patients on dialysis.^3^

SHPT manifests itself clinically in the form of pain in bones, joints, and muscles. It is also a risk factor for the occurrence of fractures, deformities, and important bone lesions, which can significantly compromise the quality of life of carriers.^4^ However, the metabolic alterations resulting from the condition considerably increase the risk of developing cardiovascular disease and progressing to death.^5^

The initial treatment of SHPT consists of adequate dialysis, a diet with control of protein intake, and the use of medications that seek to normalize the laboratory parameters and control symptoms.^6^ Between 5.5 % and 30 % of patients do not respond to conventional clinical treatment, and Parathyroidectomy (PTx) is indicated.^7^ The indication occurs if there are serum levels of PTH persistently elevated levels (> 800 pg/mL), even if not associated with symptoms and metabolic disorders.^8^^,^^9^ Even with slightly lower levels of PTH, other factors may lead to surgical indications. The procedure appears to be an important solution with much potential to improve the quality of life of people with CKD, both from the physical and psychological points of view.^9^^,^^10^ Many sick patients today are on the waiting list for surgical treatment in Brazil.^11^ Before the operation, most of them were prescribed drugs to decrease PTH secretion (calcimimetics and vitamin D analogs) or to reduce phosphorus absorption (sevelamer).

The two most commonly used techniques for the surgical treatment of SHPT are Subtotal Parathyroidectomy (S-PTx) and total Parathyroidectomy with Autograft immediate heterotopic (PTx-AG).^12^ S-PTx consists of removing almost all hyperplastic parathyroids, preserving part of one of them in its bed of origin with vascularization. This remnant is called a parathyroid stump, and the intention is to leave a volume equivalent to the size of two normal parathyroids in the same region. PTx-AG completely removes the glands from the cervical region, performing grafting of some of its fragments in the patient's heterotopic area, commonly the muscle tissue. The amount of grafted parathyroid can be estimated by weight (milligrams), but more commonly, the number and the size of the parathyroid tissue fragments are described to avoid miscalculation.^13^

Some studies have already been carried out with the aim of comparing the techniques described in terms of quality of life,^10^ biochemical parameters,^13^ and recurrence of the disease.^14^^,^^15^ Although some studies indicate that PTx-AG is more adequate for preventing recurrence, there is no consensus in the literature regarding the indication of one procedure or another, leaving the choice at the discretion of the surgeon, depending on the particularities of the patient.^16^^,^^17^

Often, it is necessary for the patient to continue using some medication after the procedure. This is due to two possible situations: the removal of the glands is not enough, keeping high levels of parathyroid hormone; or the removal of the gland is excessive and there is an important metabolic disorder, hypoparathyroidism.^18^ Both scenarios are harmful to the patient's health and are pharmacologically controlled in diverse ways, involving higher or lower costs.^19^ Although there are pre- and intraoperative measures aimed at preventing recurrences,^14^ it still affects between 10 % and 30 % of patients undergoing PTx.^20^^,^^21^

In general, PTx-AG presents lower chances of recurrence of SHPT compared to S-PTx. On the other hand, patients are more likely to develop a reverse scenario in case the removal of the gland is excessive, clinically characterized by persistent hypocalcemia with significant bone manifestations.^9^

In the unsatisfactory reduction of PTH after the operation, the patient can be reoperated or treated with drugs to try to improve the biochemical levels. Cinacalcet is an allosteric agonist of calcium-sensing receptors used to treat SHPT in patients with chronic kidney disease in an advanced stage.^6^ Its action takes place through the reduction of parathyroid hormone release and normalization of P and Ca levels.^22^ Despite its effectiveness in controlling the disease, it is an expensive drug, which makes its large-scale use a challenge, especially in developing countries.^23^ Treatment with cinacalcet is more costly for the health system, and PTx surgery itself is considered more cost-effective for the treatment of SHPT.^23^^,^^24^ In cases of recurrence or persistence after surgery, however, cinacalcet is the drug of choice for allowing satisfactory control of the condition.^25^

Likewise, sevelamer is one of the main drugs of choice to treat hyperphosphatemia. Even after a successful PTx, this dreadful condition can persist or return after bone refilling is complete. As hyperphosphatemia is independently associated with increased mortality,^11^ some patients require sevelamer after PTx. Other drugs, such as paricalcitol, calcitriol, and even medicated calcium, may be indicated for the management of metabolic factors after surgery, in certain situations.^6^

These data suggested that drug prescriptions to control metabolic imbalances after surgery would possibly differ according to the surgical procedure adopted. Consequently, the costs would be different.

In cases of reoperation, it is known that the costs could also differ according to the operative technique initially chosen. In the case of PTx-AG, the removal of the autograft requires only local anesthesia and is not only a less risky procedure but also less costly than reoperation after S-PTx.^26^^,^^27^

The main objective of the present study was to evaluate the cost of medications before and after PTx.

## Material and methods

This was a retrospective study of a cohort treated with parathyroidectomy at the Hospital das Clínicas da Universidade de São Paulo (HCFMUSP) in the period from 2012 to 2015 due to SHPT and CKD. The sample size was established for another prospective project, named RANDOMIZED CLINICAL TRIAL OF SUBTOTAL PARATHYROIDECTOMY OR TOTAL PARATHYROIDECTOMY WITH AUTOGRAFT IN CHRONIC KIDNEY DISEASE PATIENTS UNDER DIALYSIS, and detailed below.

The project aimed to detect any difference in survival between patients according to the Surgical Technique used (S-PTx or TPTx-AG). For this purpose, to detect a 30 % difference in survival after 5 years and with a loss of 15 % of patients in the follow-up, it was estimated that 41 patients would be included in three groups (namely, S-PTx, the PTx-AG with 45-fragment graft and PTx-AG with 90-fragment graft) with a power estimate of 80 % at a significance level of 5 %.

Initially, data from the patient's digital medical records were collected and included in the clinical trial. Then, those who fulfilled one or more of the exclusion criteria described below were removed. The analysis fell on the prescriptions of outpatient care that occurred after surgery. Data were analyzed from the moment of surgery up to 18 months after the procedure. For patients who presented loss of follow-up, the analysis was carried out up to the period in which they had been followed up.

The laboratory tests (with the reference ranges) analyzed were serum total Calcium (Ca) (8.6–10.2 mg/dL), serum Phosphorus (P) (2.7–4.5 mg/dL) and serum parathormone (PTH) (15–65 pg/dL).

In the review of the medical records, the description of adverse effects of the medications (e.g., nausea and vomiting for cinacalcet, hyperphosphatemia for calcitriol and paricalcitol) was verified. It was checked if there was an impossibility of treatment because of the side effects.

Then, all the medication used by the patient referring to the metabolic control of parathyroid hormone and phosphate were compiled. That analysis included the drugs cinacalcet, paricalcitol, calcitriol, sevelamer, calcium and vitamin D. The cost of medicines was estimated from data from the Ministry of Health, according to the document LISTA_CONFORMIDADE_GOV_2021_04_v1.1.pdf, available at https://www.gov.br/anvisa/pt-br/assuntos/medicamentos/cmed/precos/arquivos/lista_conformidade_gov_2021_04_v1.pdf/view. From that document, the minimum values obtained from the Maximum Sale Price to the Government (PMVG) were extracted. The costs are evaluated in Brazilian Reais (R$) as follows: Cinacalcet 30 mg (price per unity R$ 10.90), Cinacalcet 60 mg (price per unity R$ 21.32), Paracalcitol, (price per 5.0 mcg R$ 24.77), Calcitriol 0.25 mcg (price per unity R$ 1.38), Sevelamer 800 mg (price per unity R$ 2.37), and Calcium Carbonate Tablet 1,250 mg with 500 mg of calcium element (price per unity R$ 0.51). Many patients were prescribed calcium carbonate in powder form during the hungry bone phase in the early moments after PTx. The estimated cost of powder calcium carbonate per spoon was as follows: one soup spoon = R$ 6.12; dessert spoon R$ 4.08, teaspoon R$ 2.04 and coffee spoon R$ 1.02. Demographic data of age, ethnicity, and gender were analyzed. The patients were stratified by the type of operation performed. In the same periods in which the use of medications was estimated, data on total calcium, ionized calcium, phosphorus and PTH of these patients were collected.

### Inclusion criteria

Patients undergoing surgical correction of SHPT included in the RANDOMIZED CLINICAL TRIAL OF SUBTOTAL PARATHYROIDECTOMY OR TOTAL PARATHYROIDECTOMY WITH AUTOGRAFT IN CHRONIC KIDNEY DISEASE PATIENTS UNDER DIALYSIS approved by CAPPesq (CAEE 00828412.8.0000.0068) and found on Clinicaltrials.gov (NCT02464072).

### Exclusion criteria

- Patients who underwent kidney transplantation before 2 years after surgery;
- Patients who withdrew their consent throughout the study;
- Absence of prescription data in the system;
- Patients who had persistence of SHPT after operation by one gland supernumerary not identified in the initial operation. In this case, failure cannot be attributed to a specific technique and can be a confounding factor.

### Results analysis

Study data were collected and managed using REDCap electronic data capture tools hosted at Hospital das Clínicas da FMUSP.^28^^,^^29^ Statistical analysis was performed using the Graph Pad Prisma Statistical Program. Categorical variables are expressed as frequencies in absolute numbers and percentages. When relevant, they were compared with Chi-Square tests or Fisher's exact test. Continuous numerical variables were summarized by mean or median, with minimum-maximum values, Standard Deviation (±SD), or values of the first quartile (Q1) and third quartile (Q3), according to the distribution pattern analyzed by the Kolmogorov-Smirnov test. The statistical inference used a paired or unpaired *t*-test or ANOVA for normal distributions. In nonparametric distributions, the Mann-Whitney, Wilcoxon, or Kruskal-Wallis tests were used.

## Results

**Fig. 1:**
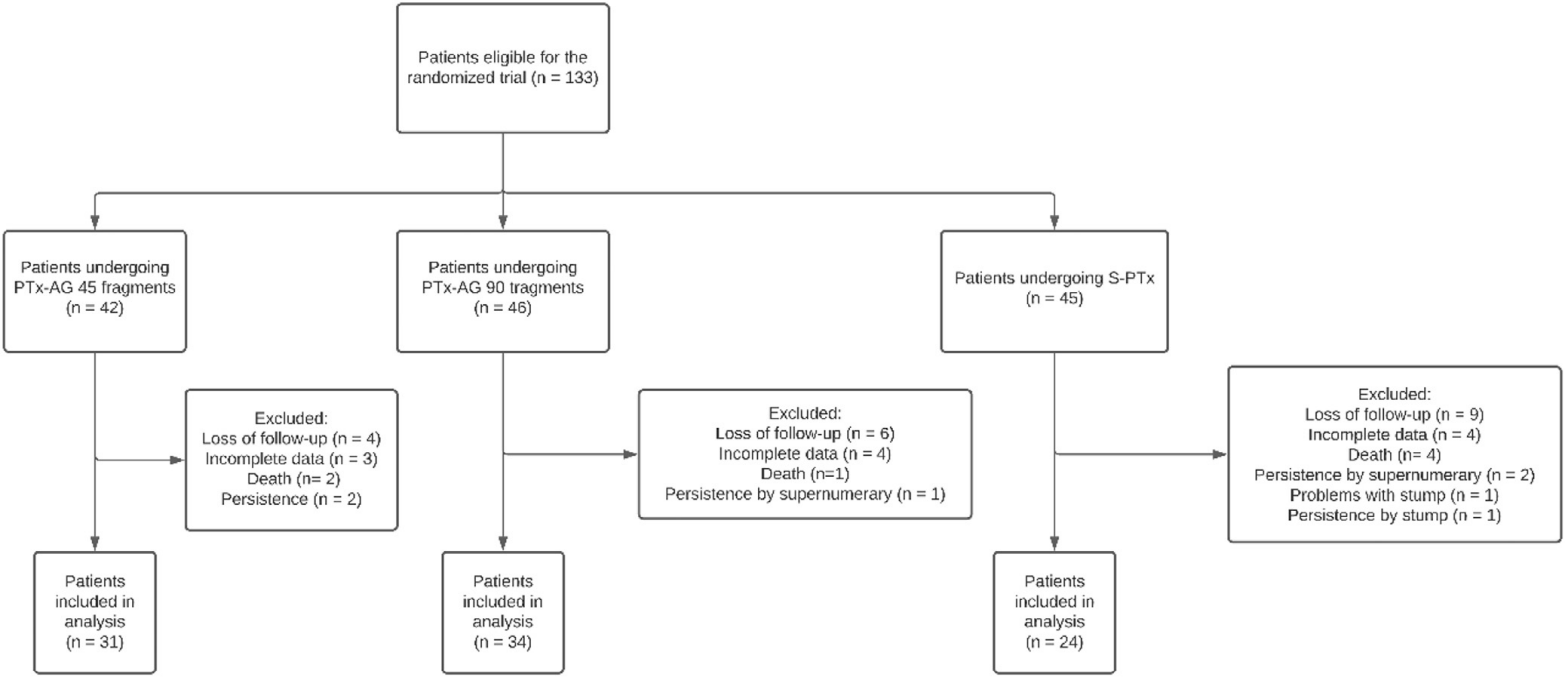
Patient allocation and causes of exclusion.

Initially, 133 patients were eligible and included for randomization after informed consent was obtained. Figure 1 shows those excluded due to loss of segment or incomplete data. Some patients died or had persistent disease due to a supernumerary gland and were also excluded. From the original cohort of the clinical trial, data from 89 patients were available for the present study.

There were 51 women, and the median (minimum‒maximum) age was 47 years (16‒68). The mean preoperative Ca was 9.8 mg/dL (minimum of 7.1 and maximum of 12.0). The median (minimum-maximum) was 1552 pg/mL (436‒7657) for Parathyroid Hormone (PTH) and 5.3 mg/dL (2.7‒10.2) for phosphorus. There was an even distribution between the male and female groups. The sex difference between each of the study arms was comparable, with some predominance of women in the 90 fragments and subtotal, but this difference was not statistically significant (Table 1).

**Table 1:** Demography and preoperative biochemical results according to groups.

	**S-PTx**	**PTx-AG 45 frag**	**PTx-AG 90 frag**	**p-value**
**N**	24	31	34	‒
**Mean Age (±SD) years**	47 (±11)	42 (±12)	44 (±14)	0.33
**Male:Female**	9:15	16:15	13:21	0.46
**Mean Calcium (±SD) mg/dL**	9.8 (±1.1)	9.8 (±0.9)	9.7 (±0.9)	0.99
**Median Phosphate (Q1-Q3) mg/dL**	5.2 (4.2‒6.5)	5.6 (5.0‒6.2)	5.0 (4.0‒6.0)	0.12
**Median PTH (Q1-Q3) (pg/mL)**	1544 (936‒1984)	1584 (1105‒1985)	1535 (1126‒2250)	0.67

**Fig. 2:**
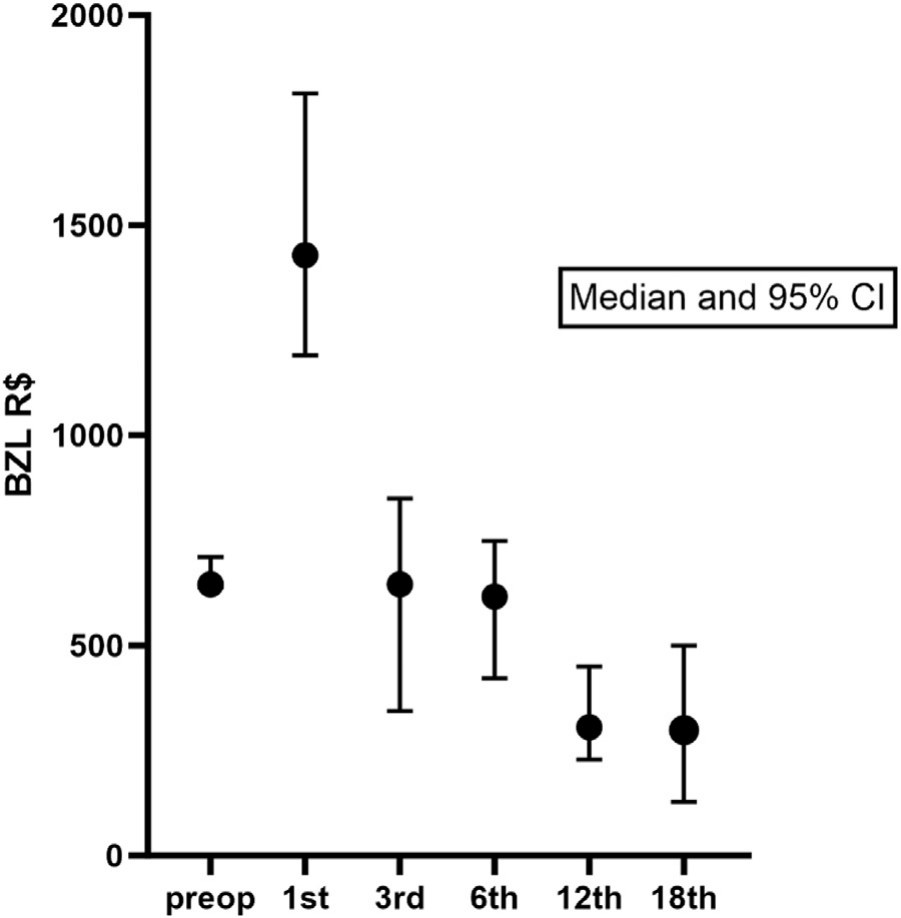
Median and 95% Confidence Interval of medication costs before and after parathyroidectomy.

Preoperatively, the median (Q1-Q3) monthly cost of medication was R$ 645.50 (429.60‒860.10). The costs more than doubled in the first month after the operation (median R$ 1429.00). At the 3^rd^ and 6^th^ postoperative time points, the monthly costs were similar to those preoperatively. After the 12^th^ month (median monthly cost of R$ 305.40), there was a sustained decrease in the costs (at 12 months p = 0.0011 and at 18 months p = 0.0015, compared to preoperative costs, Dunn's Multiple Comparisons Test). Figure 2 shows the median and the 95 % CI of medication costs pre- and postoperatively until 18 months. However, monthly costs decrease at 12 months and change little afterwards. Therefore, it is clear that after the first month, there is already a decrease in medication expenses, and from 12 months onward, these expenses remained stable.

The median of total drug costs in the analyzed period was R$ 8,375.00 (474.40‒41,703.00) per patient. There was no difference in costs per patient in the S-PTx group compared to the PTx-AG groups. Analyzing the results in month 1, month 12, and month 18, there was no difference in costs in each of the arms of the study, showing that the techniques do not impact the cost of medication in the period after surgery (Table 2).

**Table 2:** Median (Q1-Q3) monthly costs of medication postoperatively by groups.

	**S-PTx**	**PTx-AG 45 frag**	**PTx-AG 90 frag**	**p-value**
**Preoperative**	711 (569‒891)	640 (409‒806)	648 (436‒899)	0.36
1 month	1349 (740‒2590)	1238 (742‒2064)	1712 (1092‒2356)	0.50
3 months	850 (546‒1262)	724 (225‒1176)	324 (154‒881)	0.11
6 months	638 (198‒1458)	635 (298‒1366)	597 (119‒818)	0.50
12 months	444 (80‒687)	313 (192‒758)	232 (34‒586)	0.27
18 months	463 (73‒906)	424 (140‒750)	101 (12‒561)	0.06

**Fig. 3:**
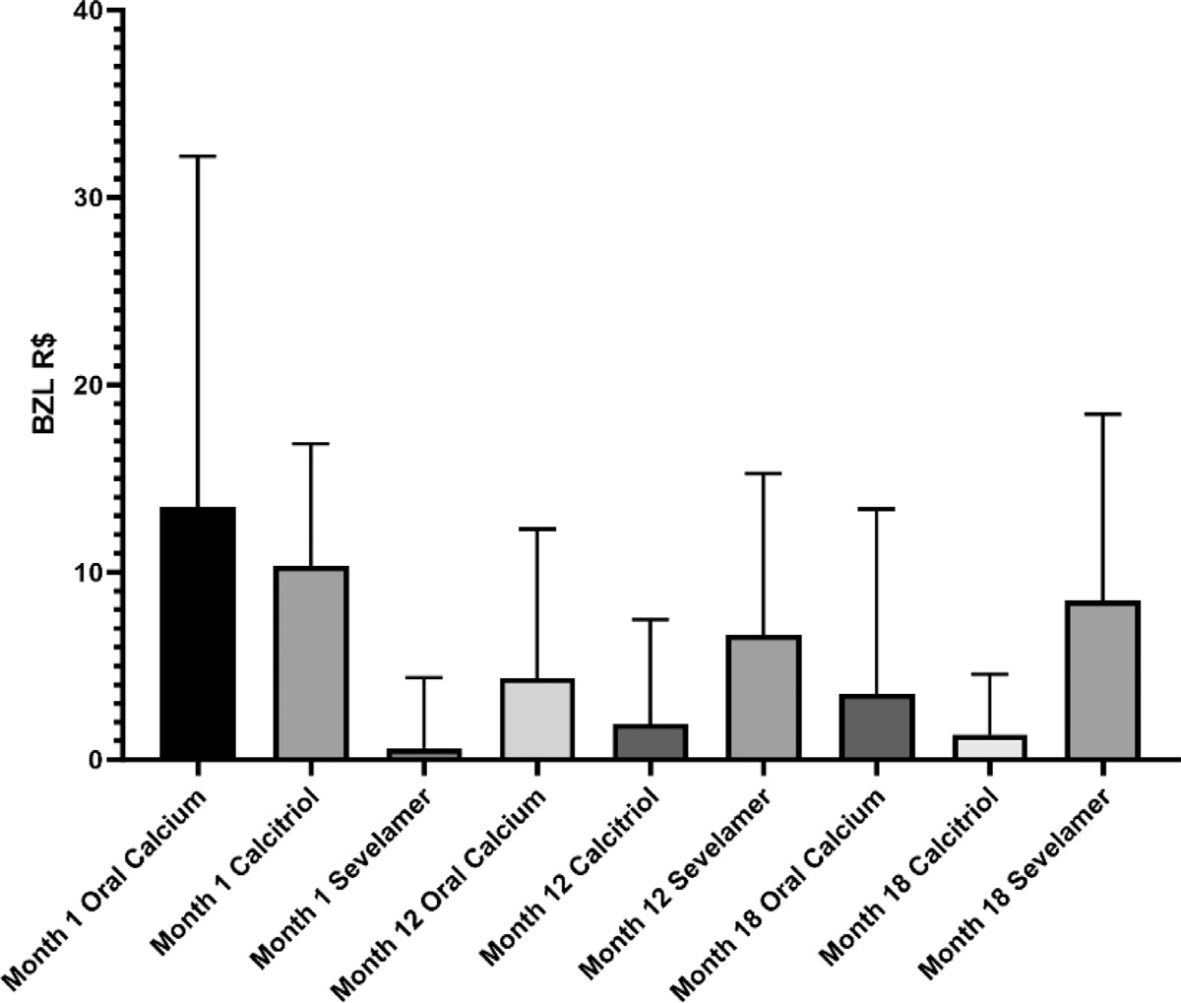
Postoperative costs of calcium, calcitriol and sevelamer at different phases.

The median cost of calcitriol in the whole group was R$ 560.0 (0‒2698) in the first month, R$ 357.4 (0‒2947) in the third month, R$ 246.3 (0‒7004) in the sixth month and 0 (0‒20038) in subsequent months. The median cost of calcium was R$ 508.4 (0‒6169) in the first month, R$ 332.9 (0‒20563) in the third month, R$ 197.3 (0‒17350) in the sixth month, R$ 178.4 (0‒8886) in the twelfth month and R$ 19.8 (0‒13164) in the eighteenth month. The cost of calcium progressively decreases, similar to the cost of calcitriol (Fig. 3).

The median cost of sevelamer was 0 (0‒2624) in the first month, 0 (0‒2588) in the third month, 0 (0‒4181) in the sixth month, 0 (0‒7082) in the twelfth month and R$ 128.0 (0‒7992) in the eighteenth month. Unlike the other medications, the cost of sevelamer, which is a phosphate binder, increases progressively, with the highest value found at 18 months. Patients who were not using sevelamer had a significantly lower cost (Fig. 3). Only one patient was prescribed cinacalcet at 12 months.

**Fig. 4:**
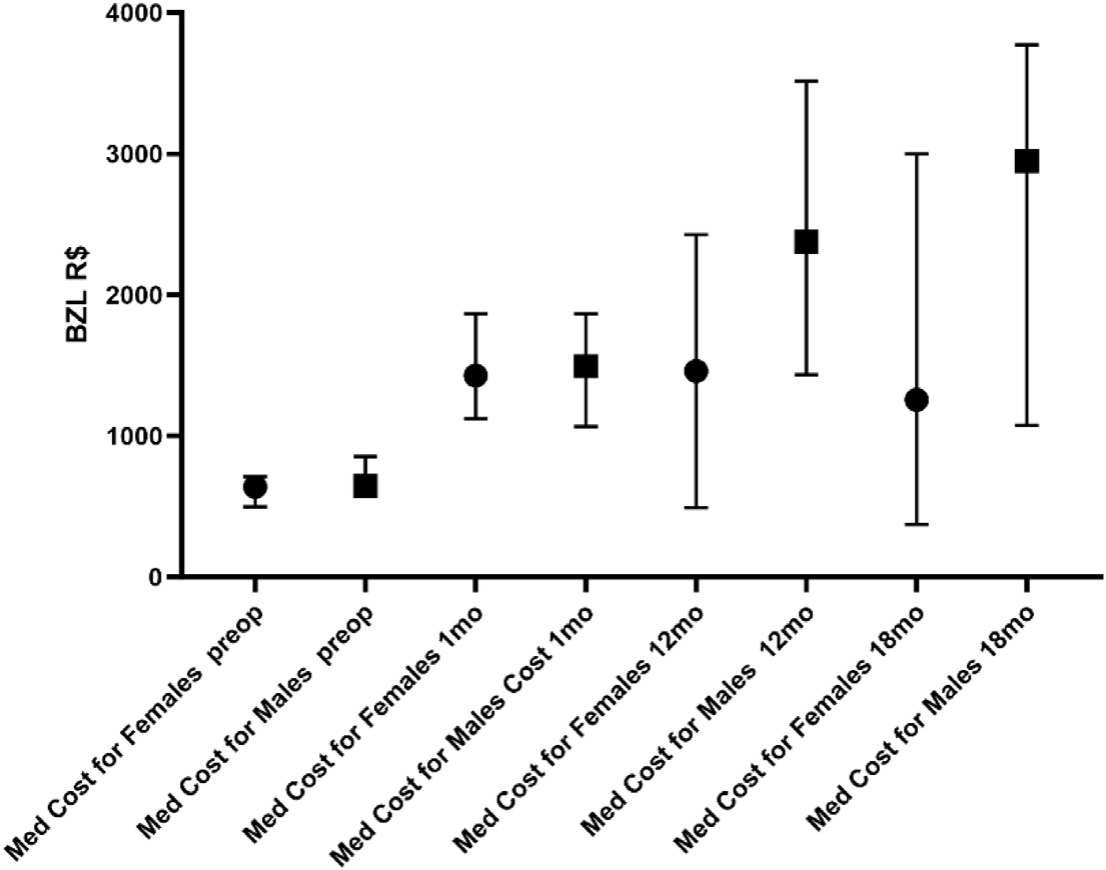
Costs of medication at selected periods according to gender.

The median total costs were R$ 11,063.0 (2322‒38748) for men and R$ 7,651.0 (474.4‒41703) for women (p = 0.0078). It is notable that men have a higher total cost than women in relation to medications (Fig. 4). The patient's gender is more important than the operation itself in determining the cost of medication. The authors also observed that men had, in practically all subgroups, a higher consumption of medication than women at 18 months.

Median preoperative PTH levels were 1869 pg/mL (639‒7657) in men and 1325 pg/mL (436‒4117) in women (p = 0.0013). A possible explanation that helps to understand why men spend more than women is that men's preoperative PTH is higher than women's. Nevertheless, there was no difference between the sexes in regard to preoperative costs.

## Discussion

The present study shows that PTx reduces the costs of medication in dialysis patients with advanced secondary hyperparathyroidism. In addition, the present data apparently show that either S-PTx or PTx-AG can reduce this cost. This finding is another important aspect to reinforce the benefits of surgery instead of medical treatment. The authors have already shown that surgery significantly improves the quality of life^10^ and even the survival rate of these patients.^30^

The present findings are in accordance with a recent study that showed a marked decrease in medication costs after parathyroidectomy. Although the costs of surgery and medical fees caused a slight increase in the net cost of the treatment to the health system, Danese et al. showed a reduction from a mean of US$ 384 to US$ 183 in the medication costs after the operation. The type of parathyroidectomy was not analyzed in that study.^31^

The present results reinforce that medication is the alternative choice only for those unable to undergo anesthesia for parathyroidectomy from an economic point of view.^32^ If improvements in long-term survival rates and quality of life are also considered, parathyroidectomy should be considered the first option, and the health system should be optimized to offer it. Otherwise, society will spend a significant part of its budget on less effective options.

The finding of a possible influence of gender on medication costs was very intriguing. There are some suggestions of gender influence on the behavior of secondary hyperparathyroidism. However, some studies have pointed out that females are more prone to develop more severe hyperparathyroidism.^33^, ^34^, ^35^, ^36^

In addition to sex, body mass index may explain the difference observed here. Perhaps patients with higher body mass may require more medication. Unfortunately, data on patient weight and height were unavailable to test this hypothesis in the present cohort.

The expectation of higher expenditure on cinacalcet in the group with subtotal parathyroidectomy due to higher levels of postoperative PTH was not confirmed. It was thought they would have higher calcium levels after surgery. However, the use of cinacalcet was observed in only one patient at 12 months and in two patients at 18 months. Therefore, there was no expressive consumption of cinacalcet by any of the study arms.

Regarding study limitations, it is important to consider that severe advanced secondary hyperparathyroidism was present in all patients. Therefore, it may not extend to secondary hyperthyroidism which does not have elevated PTH levels.

Another limitation of the study was that most patients were included when cinacalcet and paricalcitol were not available in Brazil (from 2012 to 2017). Most likely, preoperative costs may be higher currently as these medications were approved by the Ministry of Health. This point deserves another evaluation with the currently available medications and practices.

In the present analysis, the costs of reoperations (cervical or autograft excision) were not included. This question is important because cervical reoperation due to the parathyroid stump is more expensive than autograft excision under local anesthesia.

It is also relevant to consider that many patients were lost to follow-up, and it was impossible to know if they had recurrences or if they had persistent hyperparathyroidism. That is something that could impact the results. Kidney transplantation is another factor that changes the metabolic profile and impacts the analysis.

Although this was a single-center experience with very specialized teams of nephrologists and surgeons, patients were in a randomized trial and had advanced secondary hyperparathyroidism; they were all treated in a real-life scenario, following the standard pre- and postoperative protocols. Thus, the present results are probably representative of the majority of dialysis patients with PTH levels higher than 800 pg/mL and candidates for PTx.

## Conclusion

Parathyroidectomy significantly reduces medication costs eventually, despite a transient increase in the first month due to bone refilling with calcium. Either S-PTx or PTx-AG is effective in controlling severe secondary hyperparathyroidism with a reduction in the burden of medication.

## Disclaimer

The opinions, hypotheses and conclusions or recommendations expressed in the present material are under the responsibility of the authors and do not necessarily reflect the FAPESP point of view.

## Funding/financial support statement

This study was supported by grant #2021/11724-6, São Paulo Research Foundation (FAPESP).

## Declaration of competing interest

The authors declare no conflicts of interest.
